# Effects of kiwi’s pectin methylesterase inhibitor, nanomilling and pasteurization on orange juice quality

**DOI:** 10.1002/fsn3.1886

**Published:** 2020-11-11

**Authors:** Wael A. Bazaraa, Abdalla S. Ammar, Abdulghani M. Aqlan

**Affiliations:** ^1^ Department of Food Science Faculty of Agriculture Cairo University Giza Egypt

**Keywords:** juice quality, nanomilling, nano‐particles juice, orange juice, pasteurization, PME inhibitor

## Abstract

Endogenous pectin methylesterase (PME) is the enzyme responsible for phase separation and cloud loss in orange juice (OJ) manufacturing. The effect of kiwi's PME inhibitor (PMEI), nanomilling, and pasteurization on OJ quality was evaluated. The microbial quality, PME activity, OJ separation, pH, ascorbic acid content and the sensory characteristics of the juice were followed during 5 weeks storage (4°C). PMEI as freeze‐dried kiwi powder (0.3%, w/w) succeeded in inhibiting 89.3% of the OJ PME without affecting the microbial and the sensory quality. Nanomilling of OJ pulp, to prepare nano‐particles OJ (NPOJ), reduced the initial microbial load by 1.65 and 1.83 log for psychrotrophs and yeasts and molds, respectively; significantly (*p* < .05) inactivated 40.9% of the residual PME activity and the juice separation was significantly reduced by 48.3% (after 14 days of storage). Nanomilling exhibited no effect on OJ pH, but slight (*p* < .05) decrease in ascorbic acid content was noted. The combination of PMEI with NPOJ resulted in improved OJ stability with reduced separation to 36.4% of that of control. Such combination also allowed to use a lower pasteurization temperature at lower exposure time (60°C/5 min) needed to obtain new NPOJ with comparable high quality as fresh OJ and which has a shelf life of 3 weeks (4°C).

## Graphical abstract

Nanomilling was utilized for the first time in the preparation on nano‐particles orange juice. Such technique along with the use of pectin methylesterase inhibitor from kiwi improved orange juice cloud stability, improved microbial and sensory quality, as well as allowed for the use of a lower pasteurization temperature for a shorter period of times.

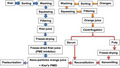

## 1 INTRODUCTION

Citrus is considered the most consumed fruits in many parts of the world especially orange, and the world production of orange in 2018 was estimated to be 75.4 million tons, while in Egypt was estimated to be 3.24 million tons (FAO, 2020). Orange fruits (Balady cultivar, *Citrus aurantium*) are very important nutritional, industrial, and commercial crop in Egypt (Khedr, 2017). Juice is the main product of orange which consumed either fresh or processed, and in both cases, the need for natural high‐quality juice with minimal or no heat treatment has considerably increased (Bull et al., 2004). Natural juices are consisting of two phases: a dispersing phase (serum) and a dispersed phase (cloud) (Mei, Hao, Zhu, Gao, & Luo, 2007). The activities of both, the spoilage microorganisms and the endogenous enzymes such as pectin methylesterase (PME), will determine the quality as well as the shelf life of the orange juice (OJ).

Pectin is considered to be the main component of the cell wall of plants and contributes to the rigidity and the integrity of plant tissues. PME (EC 3.1.1.11) catalysis the hydrolysis of the methyl group on C6 position of the polygalacturonic acid units of pectin resulting acidic pectin and methanol (Jolie, Duvetter, Van Loey, & Hendrickx, 2010). Therefore, endogenous plant PMEs have a great role in different plant development processes including stiffening, fruit ripening, and so forth. PME action can lead to two opposite effects. It can contribute to cell wall stiffens through the production of blocks of unesterified carboxyl groups which can interact with endogenous calcium ions forming pectate gel, or it can contribute to cell wall softness since proton release may stimulate other hydrolases (Giovane et al., 2004).

Juice cloud, which is composed of finely divided particles of pectin, cellulose, hemicellulose, proteins, and lipids, has an important role in the sensory characteristics of juices, that is, color, flavor, and turbidity (Agcam, Akyildiz, & Evrendilek, 2014). The premium quality juices have a stable cloud. But during manufacturing and storage of juices, cloud loss (clarification) is usually occurred causing low quality OJ (Mei et al., 2007). PME, a juice clarifying enzyme, is released into the juice during juicing process. PME de‐esterifies pectin, and the produced low methyl esterified pectin will form insoluble pectates with the reaction with calcium ions. Such insoluble gel will coprecipitate with pulp particles causing cloud loss which is considered as one of the major problems in juices industry (Iftikhar, Wagner, & Rizvi, 2014; Krall & McFeeters, 1998; Krop & Pilnik, 1974). Therefore, to produce high‐quality OJ and due to the need for minimally processed orange juice of rich flavor, PME must be inactivated (Cameron, Niedz, & Grohmann, 1994). Thermal inhibition of PME is the most common method used. However, thermal treatment negatively affects the sensory and nutritional quality of the juice (Iftikhar et al., 2014). Therefore, alternative treatments have been reported: ultrahigh pressure (Basak & Ramaswamy, 1996), high pressure (Basak, Ramaswamy, & Simpson, 2001), gamma rays (Abd‐Allah, El Kalyoubi, Abdelrashid, & Masry, 2004), pulsed electric fields (Elez‐Martinez, Suarez‐Recio, & Martin‐Belloso, 2007; Rodrigo, Barbosa‐cánovas, Martínez, & Rodrigo, 2003), recombinant PME inhibitor (PMEI) from the yeast *Pichia pastoris* (Mei et al., 2007), the proteinaceous PMEI of kiwi fruit (*Actinidia deliciosa*) (Balestrieri, Castaldo, Giovane, Quagliuolo, & Servillo, 1990; Vandevenne et al., 2011), ultrahigh pressure homogenization (Velázquez‐Estrada, Hernández‐Herrero, Guamis‐López, & Roig‐Sagués, 2012), modified super critical‐CO_2_ (Iftikhar et al., 2014), catechins, phenolics extracts from green tea leaves (L'Enfant et al., 2015; Lewis et al., 2008), thermal and combination thermal—high hydrostatic pressure (Sampedro, Rodrigo, & Hendrickx, 2008; Torres, González, Klotz, & Rodrigo, 2016), the microwave‐assisted pasteurization (Brugos, Gut, & Tadini, 2018), phenylephrine (Cheong et al., 2019), and, recently, Lactose derivatives (L'Enfant et al., 2019).

Nanotechnology is a part of science and technology that deals with the control of matter on the atomic and molecular scale—this means a size of 1 –100 nanometers. Such technology gives a wide range of food‐related applications, that is, food processing, packaging, storage, transportation, functionality, and other safety aspects of food (Bajpai et al., 2018). Nanomaterials are usually prepared by a “top‐down” (crushed into small particles using physical and/or chemical way) or “bottom‐up” (rebuilding or rearranging molecules) methods and obtain particles size in nanoscale with novel characteristics (Peters et al., 2016).

Based on the above‐mentioned considerations and since literature did not reveal the use of nanotechnology in the preparation of any nano‐juice. Therefore, the objective of this study was to prepare Nano‐Particles OJ (NPOJ) for the first time using nanomilling and to evaluate the effect of kiwi's PMEI in the reduction of pasteurization temperature of the NPOJ to obtain a higher quality juice.

## 2 MATERIALS AND METHODS

### 2.1 Materials

#### 2.1.1 Fresh fruits

Fresh Baladi orange fruits (Balady cultivar, *Citrus aurantium*) and Kiwi fruits (Green Kiwi fruit, *Actinidia deliciosa*) were purchased from local market in Giza and directly stored at 4°C until use.

#### 2.1.2 Chemicals

Citrus pectin was purchased from Sigma Chemical Company (St. Louis, Missouri, USA). Ascorbic acid and metaphosphoric acid (HPO_3_) were of analytical grade and obtained from El‐Gomhoria Co., for Chemicals trade, Cairo Egypt. Tryptic soy agar (TSA) and Potato dextrose agar (PDA) were obtained from Oxoid (England).

### 2.2 Methods

#### 2.2.1 OJ preparation

Fresh orange juice was prepared using orange juicer (Braun MPZ9 Citrus Juicer‐China). The prepared juice was then directly used after removing the seeds.

#### 2.2.2 Kiwi juice (KJ) preparation

Kiwi fruits were peeled and blended using a home type blender (Fresh, ST‐999, Egypt) for 2 min. The formed puree was filtered and pressed through cheese cloth, and the resulted clear juice was freeze‐dried (Labconco, USA).

#### 2.2.3 Proximate chemical composition

Fresh OJ and KJ juices were chemically analyzed. Moisture, protein, lipid, ash, and total carbohydrates were determined (A.O.A.C, 1990). Total carbohydrate was calculated by difference.

#### 2.2.4 pH

pH was determined using Orion (model 301) pH meter (Orion Research, USA) according to AOAC (1990).

#### 2.2.5 Ascorbic acid

Ascorbic acid was determined by HPLC as reported by Plaza et al., (2006). The analysis was carried out using an Agilent 1260 series HPLC (Agilent Technologies, California, USA). The separation was carried out using Kromasil 100–5‐C18 (4.6 × 250 mm i.d., 5 μm). The mobile phase consisted of water with 0.01% trifluoroacetic acid (pH 2.9) (A) and methanol (B) at a flow rate 1 ml/min The mobile phase was programmed with 70% A and 30% B. The multi‐wavelength detector was monitored at 262 nm. The injection volume was 10 μl for each of the sample solutions. The column temperature was maintained at 35°C.

#### 2.2.6 PME activity measurement

PME activity was measured in the fresh OJ and weekly followed in the stored juice (4°C) using the method described by Kertesz (1955).

#### 2.2.7 Separation test

Juice separation (%) was calculated as described by JBT Corporation (2018).

#### 2.2.8 The preparation of OJ with kiwi's PMEI

The kiwi's freeze‐dried powder as source of PMEI was added to the fresh OJ in the concentration range of 0.0 to 0.4%, w/w (Giovane et al., 2004). PME activity was then determined, and the best concentration that inhibited the enzyme was then used.

#### 2.2.9 The preparation of NPOJ

**Figure 1:**
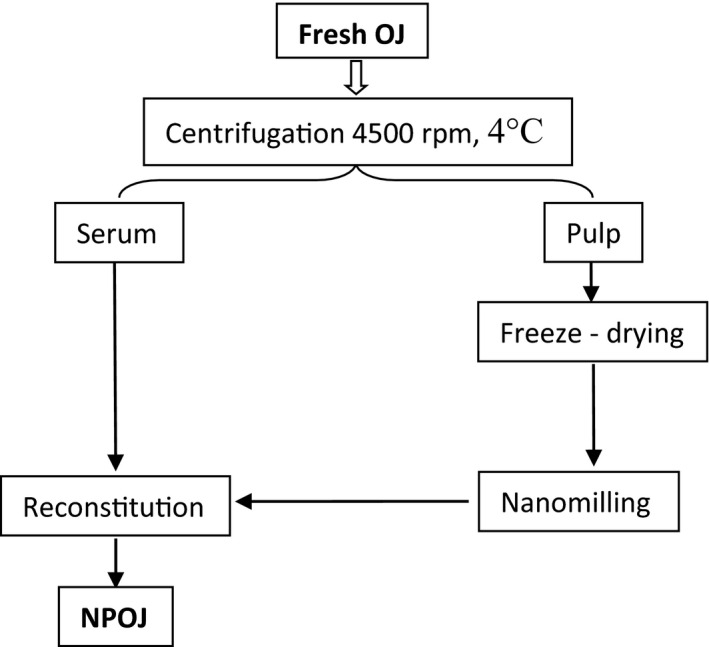
Flow diagram of the preparation of NPOJ

The NPOJ was prepared as indicated in Figure 1. Nanomilling was performed utilizing Across International PQ‐N2 Planetary Ball Mill (Gustavo, Enrique, Pablo, & Hong, 2005).

#### 2.2.10 Transmission electron microscope (TEM)

The size of the particles obtained after milling was confirmed utilizing TEM. In Eppendorf tube, approximately 50 mg of the kiwi's freeze‐dried powder (before and after nanomilling) was dissolved in 1 ml distilled water, vortexed for 5 min, and then, one drop was transferred to the top of a carbon coated grid using Pasteur pipette. After being air dried at room temperature, specimens were directly viewed utilizing Jeol TEM (JEM 1400, Japan).

#### 2.2.11 Juices pasteurization

A fresh OJ control, OJ with added kiwi powder (0.3%, w/w) (OJK), NPOJ, and NPOJ with 0.3% (w/w) kiwi powder (NPOJK) were filled in 200 ml glass bottles and heat treated in a water bath at temperature range of 50–80°C for 5 and 30 min The juices were then cooled down and kept refrigerated (4°C) for 5 weeks. The microbial quality, ascorbic acid content, pH, juice separation and PME activity were weekly followed.

#### 2.2.12 Microbial quality

Total psychrotrophic bacterial count and total yeast and mold counts were weekly followed during storage (Hatcher, Weihe, Splittstoesser, Hill, & Parish, 1992).

#### 2.2.13 Sensory quality

Organoleptic evaluation of the fresh OJ and the other prepared juices were evaluated using 15 semitrained panelists from The Food Science Department, Faculty of Agriculture, Cairo University. Samples (20 ml, each) were served in 25 ml clear plastic cups at room temperature and compared with controls. All samples were evaluated for color, odor, taste, consistency and overall acceptability on ten‐point hedonic scale, on which a score of 10 represented attributes most liked, 5 represented attributes at an unacceptable margin and 1 represented attributes most disliked (Poste, Mackie, Butler, & Larmond, 1991). Water and neutral wafers were also served for cleaning palate between samples.

### 2.3 Statistical analysis

Data were statistically analyzed using one‐way analysis of variance, ANOVA (Rao & Blane, 1985). All data were the averages of 3 experiments.

## 3 RESULTS AND DISCUSSION

### 3.1 Proximate chemical composition of OJ and KJ

The chemical composition results of both OJ and KJ are presented in Table 1. Results show that both juices varied in their composition of ash, moisture, lipids, protein, and total carbohydrates. Where OJ contained 0.21, 89.50, 0.70, 0.50 and 9.09%, while KJ contained 0.61, 83.50, 0.52, 1.16 and 14.21%, respectively. These results are in agreement with those reported by Dias et al. (2020) and Richardson, Ansell, and Drummond (2018). Results also indicated a higher content of ascorbic acid in KJ (80 mg/100 g) than that of OJ (44 mg/100 g).

**Table 1:** Chemical composition (%) of OJ and KJ

Constituent	OJ	KJ
Ash	0.21 ± 0.05	0.61 ± 0.08
Moisture	89.50 ± 0.90	83.50 ± 0.70
Total lipids	0.70 ± 0.10	0.52 ± 0.09
Total protein	0.50 ± 0.15	1.16 ± 0.18
Total carbohydrates	9.09 ± 0.60	14.21 ± 0.40
Ascorbic acid (mg/100 g)	44.00 ± 0.80	80.00 ± 0.50

### 3.2 PME activity

**Figure 2:**
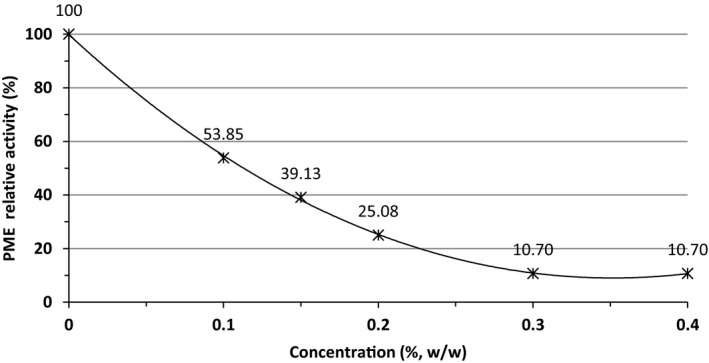
PME relative activity (%) in OJ as affected by different concentrations of freeze‐dried KJ

PMEI was originally discovered in the fully mature green kiwi fruit (Balestrieri et al., 1990; Giovane, Balestrieri, Quagliuolo, Castaldo, & Servillo, 1995). PMEI from kiwi exhibits proteinaceous nature, it acts only on plants PME, and it is not active toward other polysaccharide degrading enzymes. This protein competitively inhibits PME through the formation of a noncovalent 1:1 complex (Giovane et al., 1995). The effect of kiwi's PMEI on the orange PME activity was illustrated in Figure 2. Data (Figure 2) show that the initial PME activity in OJ was 4.49 U ml^−1^ min^−1^ (100% total activity). The addition of kiwi powder as PMEI had a significant negative effect (*p* < .05) on the activity of PME. By increasing the concentration of kiwi powder as source of PMEI, more PME inhibition was noted till reaching the maximum inhibition (89.3%) at the concentration of 0.3% (w/w) and persisted thereafter. Freeze‐dried powder of kiwi juice as a source of PMEI showed a high potential in PME inactivation at room temperature. Therefore, lower temperatures are needed for pasteurization, and as consequent, better sensory quality juice with a higher vitamin content will be expected.

**Table 2:** Effect of thermal treatments on total psychrotrophic counts of OJK during storage (4°C)

Storage (weeks)	Time (min)	Psychrotrophs				
		(log_1__0_ CFU/ml ± SD)				
		Control	50	60	70	80°C
0	5	*3.01_a_^D^ ± 0.04	2.60_b_^F^ ± 0.44	2.40_c_^F^ ± 0.10	2.08_d_^F^ ± 0.06	1.10_e_^C^ ± 0.20
	30		1.80_b_^G^ ± 0.06	1.40_c_^G^ ± 0.25	1.10_d_^G^ ± 0.04	1.01_e_D ± 0.09
1	5	4.90_a_^C^ ± 0.06	4.35_b_^D^ ± 0.18	3.90_c_^D^ ± 0.28	2.70_d_^E^ ± 0.14	1.30_e_^B^ ± 0.10
	30		3.50_b_^E^ ± 0.20	2.90_c_^E^ ± 0.18	2.08_d_^F^ ± 0.27	1.10_e_^C^ ± 0.04
2	5	6.02_a_^B^ ± 0.07	5.01_b_^C^ ± 0.36	4.50_c_^C^ ± 0.12	4.02_d_^D^ ± 0.22	1.50_e_^AB^ ± 0.08
	30		4.90_b_^CD^ ± 0.19	4.70_b_^C^ ± 0.25	4.35_c_^D^ ± 0.30	1.30_d_^B^ ± 0.10
3	5	6.40_a_^A^ ± 0.32	5.70_b_^B^ ± 0.44	5.40_c_^B^ ± 0.02	4.70_d_^CD^ ± 0.16	1.45_e_^AB^ ± 0.12
	30		5.58_b_^BC^ ± 0.29	5.10_bc_^BC^ ± 0.09	5.03_c_^C^ ± 0.14	1.30_d_^B^ ± 0.17
4	5	6.70_a_^A^ ± 0.18	6.30_b_^A^ ± 0.18	6.10_bc_^AB^ ± 0.16	5.70_c_^B^ ± 0.82	1.40_d_^AB^ ± 0.18
	30		6.20_b_^AB^ ± 0.08	6.00_bc_^AB^ ± 0.12	5.10_c_^C^ ± 0.09	1.30_d_^B^ ± 0.08
5	5	6.70_a_^A^ ± 0.18	6.45_b_^A^ ± 0.10	6.20b_c_^A^ ± 0.14	6.10_c_^A^ ± 0.02	1.70_d_^A^ ± 0.14
	30		6.36_ab_^A^ ± 0.10	6.15_b_^A^ ± 0.02	6.07_b_^A^ ± 0.20	1.45_c_^AB^ ± 0.20

* Means (±*SD*) followed by different superscripts (within columns) and different subscripts (within rows) are significantly different (*p* ≤ .05). OJK = orange juice with kiwi powder (0.3%, w/w).

**Table 3:** Effect of thermal treatments on total yeast and mold counts of OJK during storage (4°C)

Storage (weeks)	Time (min)	Yeasts & molds				
		(log_10_ CFU/ml ± SD)				
		Control	50	60	70	80°C
0	5	*2.70_a_^E^ ± 0.02	2.40_b_^E^ ± 0.18	1.80_c_^E^ ± 0.10	1.50_d_^G^ ± 0.08	1.20_e_^B^ ± 0.12
	30		1.48_b_^G^ ± 0.09	1.32_bc_^F^ ± 0.09	1.03_c_^H^ ± 0.08	0.78_d_^D^ ± 0.23
1	5	4.70_a_^D^ ± 0.10	4.30_b_^D^ ± 0.26	3.90_c_^D^ ± 0.28	3.20_d_^E^ ± 0.14	1.30_e_^AB^ ± 0.06
	30		4.01_b_^E^ ± 0.07	3.80_c_^D^ ± 0.16	1.80_d_^F^ ± 0.15	1.02_e_^C^ ± 0.28
2	5	6.10_a_^B^ ± 0.18	5.90_ab_^B^ ± 0.02	4.80_b_^C^ ± 0.12	4.40_c_^D^ ± 0.06	1.40_d_^A^ ± 0.10
	30		5.60_b_^C^ ± 0.02	4.00_c_^D^ ± 0.30	2.89_d_^E^ ± 0.26	1.06_e_^C^ ± 0.16
3	5	6.30_a_^AB^ ± 0.14	6.00_ab_^B^ ± 0.10	5.90_b_^B^ ± 0.02	5.30_c_^C^ ± 0.18	1.40_d_^A^ ± 0.16
	30		6.01_ab_^B^ ± 0.27	5.04_c_^C^ ± 0.25	3.98_d_^D^ ± 0.13	1.30_e_^AB^ ± 0.35
4	5	6.40_a_^AB^ ± 0.16	6.20_b_^A^ ± 0.24	6.01_b_^A^ ± 0.16	5.50_c_^B^ ± 0.30	1.50_d_^A^ ± 0.14
	30		6.20_b_^A^ ± 0.18	6.02_b_^A^ ± 0.07	5.40_c_^BC^ ± 0.23	1.40_d_^A^ ± 0.30
5	5	6.60_a_^A^ ± 0.18	6.30_ab_^A^ ± 0.26	6.13_b_^A^ ± 0.70	6.02_b_^A^ ± 0.03	1.50_c_^A^ ± 0.08
	30		6.30_ab_^A^ ± 0.04	6.10_b_^A^ ± 0.04	5.90_b_^AB^ ± 0.40	1.40_c_^A^ ± 0.32

* Means (±*SD*) followed by different superscripts (within columns) and different subscripts (within rows) are significantly different (*p* ≤ .05). OJK = orange juice with kiwi powder (0.3%, w/w).

### 3.3 The microbial quality of OJK as affected by heat treatments

OJK was heat treated for 5 and 30 min at 50, 60, 70 and 80°C, and the microbial quality of the juice was evaluated during 5 weeks storage at 4°C (Tables 2 and 3). Total psychrotrophic bacterial count was followed and the results (Table 2) indicated that the initial load was 3.01 log_10_ CFU/ml^,^ and this is considered of acceptable quality. Such number is much lower than that of the spoilage limit of 10^7^ CFU/ml. Also, this number did not exceed the limit of 3.7 log_10_ CFU/ml set by The GCC Standardization Organization (GSO) (2015) for fresh juices. By increasing treatment temperatures, a gradual significant (*p* < .05) decrease in psychrotrophic bacterial count was noted. Initial number decreased from 3.01 to 1.10 and 1.01 log_10_ CFU/ml after heating at 80°C for 5 and 30 min, respectively. It is of interest to report that during the first two weeks of storage there were significant differences (*p* < .05) between numbers obtained at 5 and 30 min of treatments after which no significant differences were noted. Maximum growth (6.4 log_10_ CFU/ml) was achieved after 3 weeks of storage for the control. On the other hand, the maximum growth for the heat‐treated samples was obtained at the 4th, 4th, 5th, and 2nd weeks and the maximum numbers were 6.3, 6.1, 6.1, and 1.5 log_10_ CFU/ml for samples heated for 50, 60, 70, and 80°C, respectively. From the above results, pasteurization at 80°C for 5 min was the most effective heat treatment in reduction the psychrotrophic bacterial count (1.91 log) and numbers persisted till the end of storage (1.7 log_10_ CFU/ml) below the spoilage limit. On the other hand, juice heated at 70°C was microbially accepted for only 2 weeks according to the Saudi Arabian Standards. Results (Table 3) indicated the effect of heat treatments (50–80°C) for 5 and 30 min on the total number of yeasts and molds in OJK. Similar trend for yeasts and molds was obtained as the case of psychrotrophs. By increasing temperature, a gradual decrease in numbers was noted. At zero time, numbers decreased from 2.7 to 1.2 log_10_ CFU/ml after heating for 5 min at 80°C. Heating for 30 min at the same temperature resulted in more reduction, and numbers reached 0.78 log_10_ CFU/ml. Maximum growth (6.3 log_10_ CFU/ml) for control was achieved after 3 weeks of storage, while 4, 4, 5, and 1 weeks were needed for yeasts and molds to reach maximum growth for juice heated at 50, 60, 70, and 80°C, respectively. It is of interest to report that starting from the 4th week, there were no significant differences (*p* < .05) between numbers obtained after 5 and 30 min of heat exposure. Supraditareporn and Pinthong (2007) reported a total plate count of 3.9x10^2^, 4.1x10^2^ CFU/mL at zero time and 1.8 × 10^6^, 1.9 × 10^6^ CFU/ml after 9 days of storage at 4°C for two fresh orange juices made from two different local orange varieties.

### 3.4 The effect of heat treatments of OJK on ascorbic acid content and pH level

Data (Table 4) clearly indicated the negative effect of heat on ascorbic acid content. A gradual significant (*p* < .05) loss in ascorbic acid content was noted by increasing temperature. The content was 42.5 mg/100 ml and reached 15.5 after heating OJK (control) for 5 min at 80°C with a total loss of 63.5%. Increasing heating time to 30 min resulted in an additional reduction in ascorbic acid content with a total loss 80%. On the other hand, heating at 50°C for 5 and 30 min only resulted in 26.7 and 48.6%, respectively. Supraditareporn and Pinthong (2007) reported 28.23 and 25.33 mg ascorbic acid 100^‐1^g fresh orange juices varieties Sai Nam Pung and Khieo Waan. Such concentrations were reduced to 9.93 and 8.40 mg 100g^‐1^ after 6 days of storage at 4°C, respectively. Storage for 5 weeks was less severe on the ascorbic acid content than the heat treatments. For example, no significant reduction in ascorbic acid content was noted during the first 2 weeks of storage for control and samples treated for 5 min at 50, 60, 70 and 80°C followed by minimal changes. For the control, a total loss of 29.9% was noted after 5 weeks of storage. On the other hand, a reduction of 16 and 33.4% was noted for samples heated at 80°C for 5 and 30 min, respectively. Sadecka et al. (2014) referred the reduction occurred in ascorbic acid to oxidation process taking place in juice samples during storage. Kabasakalis, Siopidou, Moshatou, and E. (2000) stated that the ascorbic acid loss was around 60 to 67% if the juices stored in open containers in a refrigerator for 31 days.

**Table 4:** Effect of thermal treatments on ascorbic acid content of OJK during storage (4°C)

Storage (weeks)	Time (min)	Ascorbic acid (mg/100 ml ± *SD*)				
		Control	50	60	70	80°C
0	5	*42.50_a_^A^ ± 0.20	31.14_b_^A^ ± 0.03	27.75_c_^A^ ± 0.20	18.97_d_^A^ ± 0.08	15.50_e_^A^ ± 0.03
	30		21.84_b_^B^ ± 0.03	19.80_c_^C^ ± 0.09	12.75_d_^C^ ± 0.09	8.50_e_^C^ ± 0.09
1	5	38.50_a_^A^ ± 0.04	30.29_b_^A^ ± 0.07	27.05_b_^A^ ± 0.24	18.19_c_^A^ ± 0.10	15.06_d_^A^ ± 0.05
	30		21.45_b_^B^ ± 0.08	18.94_c_^C^ ± 0.09	11.70_d_^C^ ± 0.05	7.51_e_^C^ ± 0.19
2	5	38.00_a_^A^ ± 0.18	29.50_b_^AB^ ± 0.02	26.34_c_^AB^ ± 0.02	18.50_d_^A^ ± 0.02	14.50_e_^AB^ ± 0.20
	30		20.03_b_^C^ ± 0.09	16.90_c_^CD^ ± 0.05	11.54_d_^C^ ± 0.03	7.50_e_^C^ ± 0.03
3	5	31.90_a_^B^ ± 0.28	28.30_b_^AB^ ± 0.08	25.33_bc_^B^ ± 0.15	16.90_c_^B^ ± 0.15	14.10_d_^AB^ ± 0.05
	30		19.45_b_^C^ ± 0.18	15.80_c_^D^ ± 0.20	10.02_d_^D^ ± 0.16	7.20_e_^C^ ± 0.07
4	5	30.83_a_^B^ ± 0.30	24.02_b_^B^ ± 0.05	20.83_c_^C^ ± 0.09	14.78_d_^B^ ± 0.07	13.50_d_^B^ ± 0.2
	30		19.01_b_^C^ ± 0.22	15.40_c_^D^ ± 0.01	9.80_d_^D^ ± 0.23	7.04_e_^C^ ± 0.26
5	5	29.80_a_^B^ ± 0.28	23.09_b_^B^ ± 0.15	20.26_b_^C^ ± 0.04	15.32_c_^B^ ± 0.14	13.02_d_^B^ ± 0.23
	30		18.50_b_^C^ ± 0.06	15.03_c_^D^ ± 0.23	9.30_d_^D^ ± 0.03	5.66_e_^D^ ± 0.04

* Means (±*SD*) followed by different superscripts (within columns) and different subscripts (within rows) are significantly different (*p* ≤ .05). OJK = orange juice with kiwi powder (0.3%, w/w).

Increasing the temperature up to 80°C seemed to have very little impact on pH values of the OJK (Table 5). Also, increasing heating time from 5 to 30 min showed no effect at all. However, a little but significant reduction was noted during storage and this was probably due to the microbial activities. The lowest obtained pH values were 3.54 (control, 2 weeks), 3.55 (50°C, 2 weeks), 3.53 (60°C, 4 weeks), 3.54 (70°C, 4 weeks), and 3.56 (80°C, 4 weeks) for both samples treated for 5 and 30 min Dewanti‐Hariyadi (2013) referred the low pH of many fruits and their juices due to their high organic acid content. Likewise, Aneja, Dhiman, Aggarwal, Kumar, and Kaur (2014) reported a pH range of 4.19 to 4.5 for orange juice. Also, Villamiel, Dolores del Castillo, Martin, and Corzo (1998) stated that the pH value of 3.61of orange juice (Navel variety) remained without change during heat treatment.

**Table 5:** Effect of thermal treatments on pH level of OJK during storage (4°C)

Storage (weeks)	Time (min)	pH ± *SD*				
		Control	50	60	70	80°C
0	5	*3.67_b_^A^ ± 0.18	3.68_b_^A^ ± 0.08	3.69_b_^A^ ± 0.22	3.70_ab_^A^ ± 0.07	3.74_a_^A^ ± 0.09
	30		3.69_b_^A^ ± 0.09	3.70_ab_^A^ ± 0.15	3.71_a_^A^ ± 0.20	3.75_a_^A^ ± 0.23
1	5	3.60_c_^A^ ± 0.14	3.62_b_^A^ ± 0.07	3.64_b_^A^ ± 0.08	3.65_ab_^A^ ± 0.06	3.68_a_^A^ ± 0.05
	30		3.65_b_^A^ ± 0.16	3.65_b_^A^ ± 0.20	3.66_ab_^A^ ± 0.10	3.70_a_^A^ ± 0.43
2	5	3.54_c_^B^ ± 0.20	3.55_b_^B^ ± 0.05	3.57_ab_^A^ ± 0.05	3.59_a_^A^ ± 0.13	3.61_a_^A^ ± 0.08
	30		3.58_c_^B^ ± 0.19	3.59_bc_^A^ ± 0.14	3.63_b_^A^ ± 0.24	3.69_a_^A^ ± 0.13
3	5	3.52_b_^B^ ± 0.08	3.53_b_^B^ ± 0.04	3.55_ab_^A^ ± 0.14	3.56_ab_^A^ ± 0.08	3.59_a_^A^ ± 0.20
	30		3.57_b_^B^ ± 0.28	3.59_ab_^A^ ± 0.30	3.60_ab_^A^ ± 0.06	3.63_a_^A^ ± 0.15
4	5	3.50_b_^B^ ± 0.03	3.52_ab_^B^ ± 0.14	3.53_ab_^B^ ± 0.22	3.54_a_^B^ ± 0.19	3.56_a_^B^ ± 0.12
	30		3.54_a_^B^ ± 0.10	3.54_a_^B^ ± 0.05	3.56_a_^B^ ± 0.18	3.57_a_^B^ ± 0.19
5	5	3.50_b_^B^ ± 0.04	3.50_b_^B^ ± 0.19	3.53_ab_^B^ ± 0.15	3.54_a_^B^ ± 0.15	3.56_a_^B^ ± 0.18
	30		3.51_b_^B^ ± 0.17	3.52_ab_^B^ ± 0.13	3.53_ab_^B^ ± 0.12	3.56_a_^B^ ± 0.26

* Means (±*SD*) followed by different superscripts (within columns) and different subscripts (within rows) are significantly different (*p* ≤ .05). OJK = orange juice with kiwi powder (0.3%, w/w).

### 3.5 PME activity as affected by heat treatments

Table 6 indicates the relationship between heating OJK at 50, 60, 70 and 80°C for 5 and 30 min and PME activity. Data clearly revealed significant (*p* < .05) loss in PME activity by increasing temperature. At zero time, the activity was 0.48 for the control and reached 0.30 U ml^‐1^ min^‐1^ after 5 min of heating at 80°C with a total loss of 37.5%. Increasing exposure to 30 min increased the total loss to 54.2%. Since PMEI originally inhibited 89.3% of the original PME activity, therefore, a total loss of 93.31 and 95.1% in PME activity was achieved due to the use of PMEI (0.3%, w/w) in combination with heating at 80°C for 5 and 30 min, respectively. Significant (*p* < .05) differences between the activities obtained at 5 and 30 min were found in the case of heating at 70 and 80°C while it was absent at 50 and 60°C. Such PME residual activities are probably due to the presence of different PME isoforms some of which are extremely thermostable (Jolie et al., 2010; La Ratta et al., 1995). The PME‐PMEI complex will not dissociate at high temperature but rather denature as a single entity (Jolie et al., 2010). During storage, PME activity showed varied degrees of increase depending on the used temperature. Potential PME reactivation had been reported, where Balaban et al. (1991) and Iftikhar et al. (2014) indicated PME reactivation in OJ during storage at 4°C. Also, Guerrero‐Beltran, Barbosa‐Cánovas, and Swanson (2004) and Welti‐Chanes, Ochoa‐Velasco, and Guerrero‐Beltrán (2009) reported an increase in PME activity during storage of OJ at 4°C due to isoenzymes arising. Other studies (Elez‐Martinez, Solvia‐Fortuny, & Martin‐Belloso, 2006; Rivas, Rodrigo, Barbosa‐Cánovas, Martínez, & Rodrigo, 2006; Salvia‐Trujillo, Morales‐de la Peña, Rojas‐Graü, & Martín‐Belloso, 2011) reported a constant PME activity during the storage of the heat pasteurized OJ. On the other hand, the irreversible inactivation of PME (the activity was maintained or decreased during storage) in OJ was also reported (Agcam et al., 2014; Elez‐Matrinez et al., 2006; Sampedro, Geveke, Fan, & Zhang, 2009; Vervoort et al., 2011).

**Table 6:** Effect of thermal treatments on PME activity of OJK during storage (4°C)

Storage (weeks)	Time (min)	PME (U ml^−1^ min^−1^ ± *SD*)				
		Control	50	60	70	80°C
0	5	*0.48_a_^E^ ± 0.08	0.44_b_^D^ ± 0.34	0.38_c_^F^ ± 0.12	0.34_d_^E^ ± 0.30	0.30_e_^D^ ± 0.08
	30		0.42_b_^D^ ± 0.12	0.36_c_^F^ ± 0.16	0.30_d_^F^ ± 0.22	0.22_e_^E^ ± 0.09
1	5	0.72_a_^D^ ± 0.06	0.63_b_^C^ ± 0.12	0.52_c_^E^ ± 0.04	0.48_d_^CD^ ± 0.20	0.42_e_^BC^ ± 0.10
	30		0.58_b_^C^ ± 0.16	0.46_c_^EF^ ± 0.13	0.34_d_^E^ ± 0.10	0.26_e_^DE^ ± 0.16
2	5	0.98_a_^C^ ± 0.10	0.74_b_^B^ ± 0.08	0.63_c_^D^ ± 0.08	0.56_d_^C^ ± 0.07	0.50_e_^B^ ± 0.14
	30		0.78_b_^B^ ± 0.19	0.62_c_^D^ ± 0.19	0.40_d_^D^ ± 0.23	0.30_e_^D^ ± 0.12
3	5	1.18_a_^BC^ ± 0.12	0.96_ab_^AB^ ± 0.04	0.84_b_^C^ ± 0.26	0.72_c_^B^ ± 0.06	0.58_d_^AB^ ± 0.20
	30		0.98_ab_^AB^ ± 0.17	0.78_b_^CD^ ± 0.11	0.45_c_^CD^ ± 0.12	0.36_d_^C^ ± 0.15
4	5	1.22_a_^B^ ± 0.14	1.04_a_^AB^ ± 0.02	0.96_b_^AB^ ± 0.06	0.78_bc_^A^ ± 0.02	0.62_c_^A^ ± 0.16
	30		1.00_ab_^AB^ ± 0.24	0.92_b_^B^ ± 0.09	0.50_c_^C^ ± 0.15	0.36_d_^C^ ± 0.31
5	5	1.30_a_^A^ ± 0.26	1.08_ab_^A^ ± 0.08	1.00_ab_^A^ ± 0.22	0.80_b_^A^ ± 0.24	0.64_c_^A^ ± 0.12
	30		1.10_b_^A^ ± 0.09	1.02_b_^A^ ± 0.10	0.52_c_^C^ ± 0.60	0.38_d_^C^ ± 0.24

* Means (±*SD*) followed by different superscripts (within columns) and different subscripts (within rows) are significantly different (*p* ≤ .05). OJK = orange juice with kiwi powder (0.3%, w/w).

**Figure 3:**
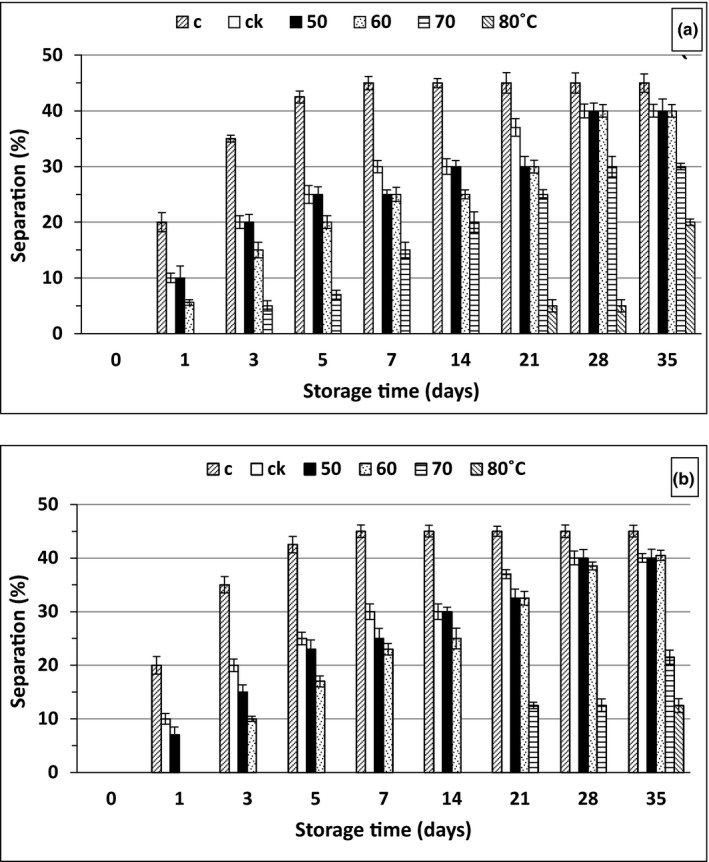
(a,b) Effect of different heat treatments on the separation (%) of OJK during cold storage (4°C) where (a) represents 5 min treatment and (b) represents 30 min

Juice separation is considered as a major defect in juice industry and occurred as a direct effect of PME. Figure 3a,b indicates the effect of pasteurization at 50, 60, 70 and 80°C for 5 (3a) and 30 min (3b) on the OJK separation (%) during cold storage for 5 weeks. At zero time, separation was absent in all samples and started to appear from day 1 in most of the samples at various rates. For samples treated for 5 min, the control reached the maximum separation (42.5%) at the 5th day of storage with no significant differences thereafter. Separation phenomenon is generally referred to PME activity. Therefore, the addition of 0.3% kiwi powder as source of PMEI resulted in 50% reduction in separation after 1 day of storage. In general, increasing treatment temperature resulted in lower separation values. There were no significant differences between values for control with added kiwi and those treated at 50°C. This was obviously due to the thermostability of PME at this temperature and the reduction in separation values was only due to the effect of the inhibitor. Higher temperatures negatively affected the PME activity, and as a consequent, significant (*p* < .05) reduction in separation values appeared till reaching its maximum value at 80°C. At 80°C (5 min), OJK started to show up after 21 days of the cold storage. Similar trend was noticed with samples treated for 30 min but with higher reduction in separation values. Best results were obtained at 80°C followed by 70°C, where separation in OJK started to appear after 35 and 21 days, respectively, with a value of 12.5% for both. On the other hand, separation started to appear after 21 days in case of samples treated at 80°C for 5 min with a value of 5.0%. PME inactivation of 84% was obtained when the orange juice was treated at 90°C/60s (Iftikhar et al., 2014), while Kim, Tadini, and Singh (1999) described 100% inactivation at 80°C/70s. Also, SentandreuCarbonell, Carbonell, and Izquierdo (2005) found that PME residual activities in thermally treated juices from oranges, mandarins, and hybrids were 0, 1, 3, 15, and 20% at 95°C/20s, 90°C/10s, 85°C/10s, 80°C/5‐20s, and 70°C /5‐20s, respectively. Recently, Brugos et al. (2018) reported that at temperatures between 85.0 and 95.0°C/5s, the PME inactivation reached 95.3 to 97.5%.

### 3.6 Nano‐particles orange juice (NPOJ)

**Figure 4:**
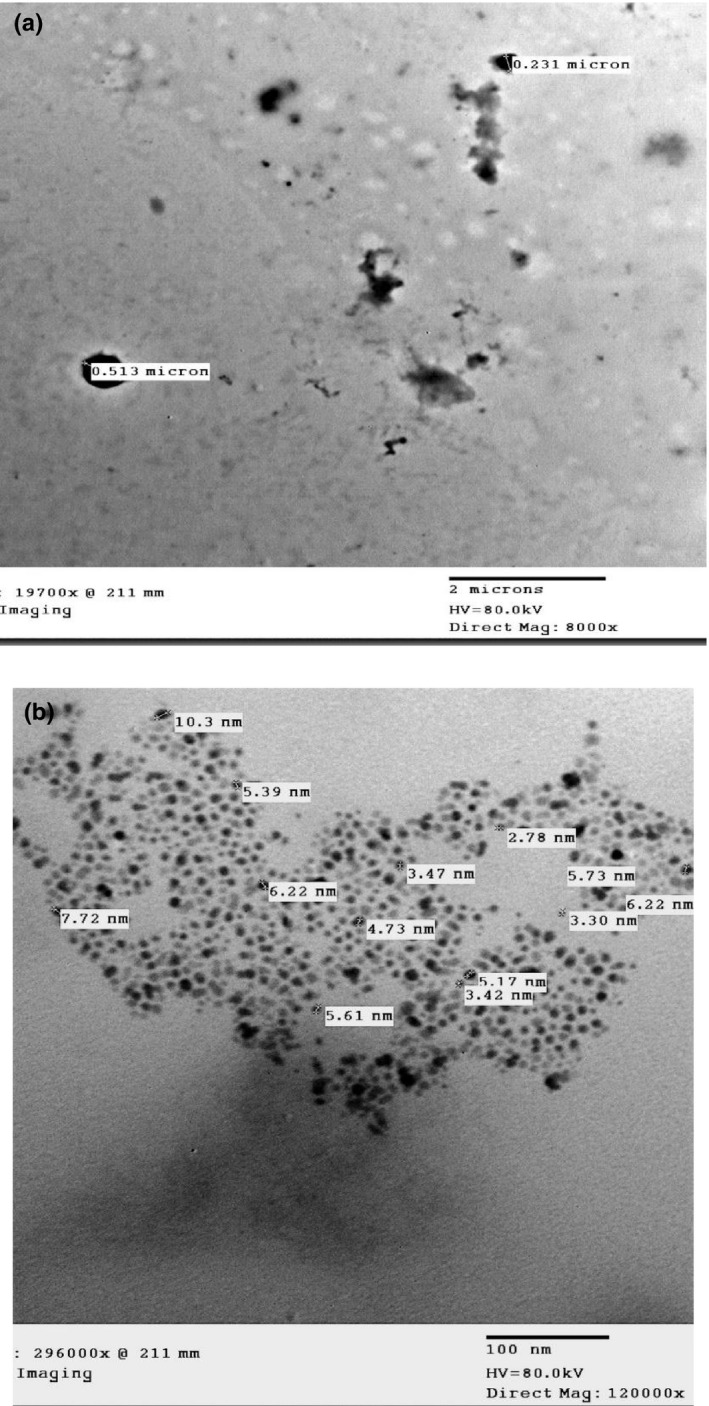
(a) TEM image OJ cloud particles prior to nanomilling. (b) TEM image of OJ cloud particles after nanomilling

As indicated earlier, nanotechnology means the conversion of macromolecules to smaller size particles with a size range of 1–100 nm to get new materials with improved characteristics. Figure 4a,b shows TEM images of OJ biomolecules prior to (a) and after (b) nanomilling. Prior to nanomilling (Figure 4a), OJ molecules particle size ranged from 0.231 to o.513 µm with the presence of high cell debris. Croak and Corredig (2006) stated that OJ cloud particles size ranged from 400 to 5,000 nm. They also indicated that particles with a size smaller than 2 µm are generally stable. On the contrary, Leizerson and Shimon (2005) reported 188.8 µm as an average size of the OJ cloud particle. Figure 4b indicates the size of OJ biomolecules after nanomilling. The resulted particles had a size range of 2.5 to 10.3 nm which assured good milling process.

After the reconstitution of OJ, the quality characteristics (microbial, PME activity, ascorbic acid, and pH) were evaluated for 2 weeks at 4°C and the results were presented in Table 7. The initial number of total psychrotrophs in OJ and NPOJ was 3.28 and 1.63 log_10_ CFU/ml, respectively. During the storage, numbers increased to 6.02 and 5.37 log_10_ CFU/ml for OJ and NPOJ, respectively. Similarly, yeast and mold numbers were 3.44 and 1.61 for OJ and NPOJ at zero time, respectively. Numbers increased during storage reaching 6.15 and 5.72 log_10_ CFU/ml, respectively. It is of interest to report that all obtained counts for NPOJ were lower (*p* < .05) than those of OJ. Such significant reduction was probably due to direct destruction of cells by the nanomilling process.

**Table 7:** The effect of nanomilling on microbial quality, PME activity, ascorbic acid content and pH of the NPOJ during storage (2 weeks at 4°C)

Storage (weeks)	Sample	Psychrotrophs (log_10_CFU ml^−1^)	Y&*M* (log_10_CFU ml^−1^)	PME (U ml^−1^ min^−1^)	Ascorbic acid (mg/100 ml)	pH
0	OJK	*3.28^D^ ± 0.10	3.44^D^ ± 0.16	0.44^A^ ± 0.07	33.79^A^ ± 0.15	3.74^A^ ± 0.20
	NPOJK	1.63^E^ ± 0.18	1.61^E^ ± 0.30	0.26^C^ ± 0.14	28.50^B^ ± 0.24	3.71^A^ ± 0.12
1	OJK	4.59^C^ ± 0.02	5.68^B^ ± 0.10	0.43^A^ ± 0.09	31.64^A^ ± 0.25	3.56^B^ ± 0.16
	NPOJK	3.46^D^ ± 0.14	4.02^C^ ± 0.23	0.26^C^ ± 0.02	26.99^B^ ± 0.12	3.68^A^ ± 0.10
2	OJK	6.02^A^ ± 0.18	6.15^A^ ± 0.40	0.35^B^ ± 0.12	31.41^A^ ± 0.26	3.41^C^ ± 0.09
	NPOJK	5.37^B^ ± 0.06	5.72^B^ ± 0.17	0.21^C^ ± 0.05	24.76^C^ ± 0.33	3.55^B^ ± 0.22

Note

OJK = orange juice with kiwi powder (0.3%, w/w), NPOJK = nano‐particles orange juice with kiwi powder (0.3%, w/w).

* Means (±*SD*) followed by different superscripts within each column are significantly different (*p* < .05).

Results (Table 7) indicate that nanomilling resulted in 40.9% reduction in the initial PME activity, and this may be due to the partial destruction of the enzyme by the effect of direct milling. After storage for 1 and 2 weeks, such reduction was almost constant with no change. It could be concluded that PMEI inhibited 89.3% of PME activity followed by an additional 40.9% loss of the remaining activity (10.7%) due to nanomilling. Therefore, a total loss of 93.7% of the PME activity was achieved.

**Figure 5:**
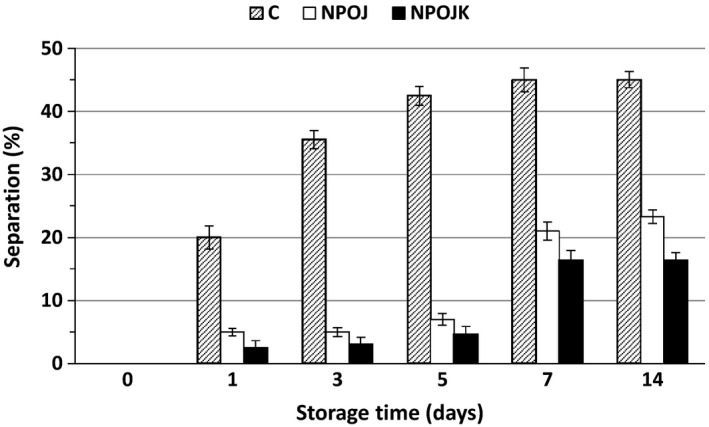
Effect of nanomilling and PMEI on the separation (%) of OJ during storage at 4°C. C represents control, NPOJ represents nano‐particles orange juice and NPOJK represents nano‐particles orange juice with added kiwi's freeze‐dried powder (0.3%, w/w)

The effect of nanomilling on the percentage of juice separation of the NPOJ is shown in Figure 5. Nanomilling significantly (*p* < .05) reduced separation by 75.0, 84.0, 83.5, 53.3 and 48.3% compared with control during cold storage for 1, 3, 5, 7 and 14 days, respectively. Such reduction in separation percentage was due to the direct mechanical destruction effect of nanomilling on PME as well as the significant reduction in OJ particles size to the nano size which gave more stable juice (Croak & Corredig, 2006). Many authors also stated that the cloud stability of the treated orange juice depends not only on PME inactivation but is also affected by the changes in pectin structure and size reduction of cloud particles (Lacroix, Fliss, & Makhlouf, 2005; Tiwari, Muthukumarappan, O'Donnell, & Cullen, 2009). The addition of PMEI to the NPOJ positively improved the stability of the juice by partially inhibiting the remaining activity of PME where the range of the remaining separation (%) was 50.0 to 78.1% of that obtained with NPOJ without the addition of the inhibitor.

Table 7 also indicates a constant content of ascorbic acid during 2 weeks of storage of OJ. On the other hand, slight but significant (*p* < .05) decrease in ascorbic acid content was noted at zero time (15.7%) and after 2 weeks (26.7%) of storage. Nanomilling showed no effect on the pH level of OJ (Table 7). At zero time, the pH of the OJ and NPOJ was the same and this assures that such milling had no effect on pH. During storage, pH values became lower (*p* < .05) specially in case of OJ and the values were 3.56 and 3.41 for juice stored for 1 and 2 weeks, respectively. Such changes were due to the microbial activities in juice during storage producing acids.

### 3.7 Sensory evaluation

Results of sensory evaluation of OJK and NPOJK heat treated at 50, 60, 70 and 80°C for 5 and 30 min compared to fresh OJ control are summarized in Table 8. First, the addition of the kiwi's freeze‐dried powder (0.3%, w/w) had no significant (*p* < .05) effect on any of the tested sensory parameters. On the other hand, nanomilling resulted in little but significant reduction in all tested parameters. For example, NPOJ showed lower taste scores and such low scores were obtained since the juice exhibited a bitter taste. In OJ, bitterness is mainly referred to the presence of limonin and naringin. Generally, an increase in bitterness is noted in juices of some varieties after extraction (delayed bitterness), which is considered to be a major industrial problem. The major cause of the delayed bitterness is limonin, and the threshold for limonin is 6 ppm in orange juice at pH 3.8. Limonin is produced by the conversion of the nonbitter precursor, limonoate A‐ring lactone, to limonin, by the enzyme limonin D‐ring lactone hydrolase, under acidic conditions (Ribeiro, Silveria, & Ferreira‐Dias, 2002). The increase in bitterness was probably due to the effect of nanomilling in accelerating the synthesis of limonin. Siddiqui, Kulkarni, Kulkarni, and Mulla (2013) indicated that pasteurization increased limonin content to 152 mg/L which resulted in the bitterness of the juice. Nanomilling negatively affected juice color, and this was probably due to the partial oxidation of carotenoids responsible of juice color (Cinquanta, Albanese, Cuccurullo, & DiMatteo, 2010). The reduction noted in odor scores was probably due to the partial loss in orange essential oil due to milling of the freeze‐dried orange pulp. Also, the reduction in particle size negatively affected the consistency. Increasing both of pasteurization temperature and treatment time significantly (*p* < .05) resulted in a sharp decline in the studied sensory characteristics where higher rates of oxidation and browning took place, and consequently, color, taste, and odor were negatively affected (Deterre et al., 2016). For example, increasing the temperature from the room temperature for the NPOJ control to 50°C (5 min) resulted in 1.5% loss in taste scores. Elevation the temperature by additional 20°C (50–70°C) resulted in 8.4% total loss in taste scores, while increasing the temperature from 70 to 80°C led to the highest loss in taste scores (23.7%). Taste was the most sensitive characteristic affected by heat. Also, 30 min treatment at 80°C was responsible for the worst juice. The sensory scores were declining by increasing temperature, and the lowest scores were for samples heat treated at 80°C. In general, scores recorded for 30 min treatments were lower than those of 5 min The best tested heat treatment was 60°C for 5 min where the overall acceptability scores were the same as control OJ with no significant differences. It is of interest to indicate that scores obtained at 60°C for 5 min were higher than those obtained at 50°C for 30 min

**Table 8:** Sensory evaluation of OJK and NPOJK pasteurized at different temperatures for 5 and 30 min

Treatment	Taste	Color	Odor	Consistency	Overall acceptability
OJ	*9.90_a_ ± 0.20	9.85_a_ ± 0.30	9.95_a_ ± 0.52	9.89_a_ ± 0.60	9.90_a_ ± 0.16
OJK**	9.80_a_ ± 0.38	9.72_a_ ± 0.14	9.92_a_ ± 0.16	9.68_a_ ± 0.46	9.86_a_ ± 0.10
NPOJK	9.64_b_ ± 0.14	9.48_b_ ± 0.40	9.50_b_ ± 0.36	9.24_b_ ± 0.12	9.32_b_ ± 0.24
50°C /5m	9.66_b_ ± 0.22	9.71_a_ ± 0.45	9.84_a_ ± 0.52	9.55_a_ ± 0.37	9.72_a_ ± 0.43
	(9.50_b_ ± 0.34)	(9.35_b_ ± 0.27)	(9.50_b_ ± 0.60)	(9.20_b_ ± 0.65)	(9.52_ab_ ± 0.30)
50°C /30m	9.15_bc_ ± 0.29	9.44_b_ ± 0.18	9.75_a_ ± 0.82	9.52_ab_ ± 0.39	9.50_ab_ ± 0.14
	(8.81_d_ ± 0.42)	(9.32_b_ ± 0.20)	(9.42_b_ ± 0.24)	(9.15_bc_ ± 0.64)	(9.10_c_ ± 0.44)
60°C /5m	9.35_bc_ ± 0.33	9.53_b_ ± 0.54	9.70_a_ ± 0.43	9.53_ab_ ± 0.19	9.60_a_ ± 0.53
	(9.27_bc_ ± 0.13)	(9.44_b_ ± 0.24)	(9.38_b_ ± 0.31)	(9.30_b_ ± 0.44)	(9.51_ab_ ± 0.28)
60°C /30m	8.22_d_ ± 0.12	9.10_c_ ± 0.37	9.62_b_ ± 0.45	8.93_bc_ ± 0.62	9.00_c_ ± 0.41
	(7.95_e_ ± 0.29)	(8.78_c_ ± 0.61)	(9.31_c_ ± 0.36)	(8.70_bc_ ± 0.34)	(8.78_c_ ± 0.23)
70°C /5m	8.96_c_ ± 0.12	9.34_b_ ± 0.30	9.60_b_ ± 0.10	9.48_b_ ± 0.14	9.40_b_ ± 0.42
	(8.70_d_ ± 0.54)	(9.22_b_ ± 0.30)	(9.44_b_ ± 0.10)	(9.16_bc_ ± 0.48)	(9.06_c_ ± 0.50)
70°C /30m	7.10_f_ ± 0.50	8.50_c_ ± 0.16	8.36_c_ ± 0.64	8.54_c_ ± 0.10	8.72_c_ ± 0.58
	(6.90_f_ ± 1.18)	(8.26_cd_ ± 0.82)	(8.22_cd_ ± 0.56)	(8.14_d_ ± 0.18)	(8.24_cd_ ± 0.36)
80°C /5m	6.76_f_ ± 0.24	8.18_cd_ ± 0.08	8.10_d_ ± 0.12	8.28_cd_ ± 0.34	8.50_c_ ± 0.20
	(6.64_f_ ± 0.70)	(8.14_d_ ± 0.46)	(8.06_d_ ± 0.78)	(8.10_d_ ± 0.24)	(8.08_d_ ± 0.62)
80°C /30m	5.04_g_ ± 0.10	5.68_e_ ± 0.42	5.40_e_ ± 0.22	6.12_e_ ± 0.22	5.56_e_ ± 1.10
	(4.78_g_ ± 1.32)	(5.08_f_ ± 1.10)	(4.96_f_ ± 0.38)	(5.40_f_ ± 1.22)	(5.14_f_ ± 1.14)

* Means (±*SD*) followed by different subscripts within each column are significantly different (*p* ≤ .05). Means between parenthesis are for the NPOJK.

** OJK = orange juice with added kiwi's freeze‐dried powder (0.3%, w/w), NPOJK = nano‐particles orange juice with added kiwi's freeze‐dried powder (0.3%, w/w).

**Figure 6:**
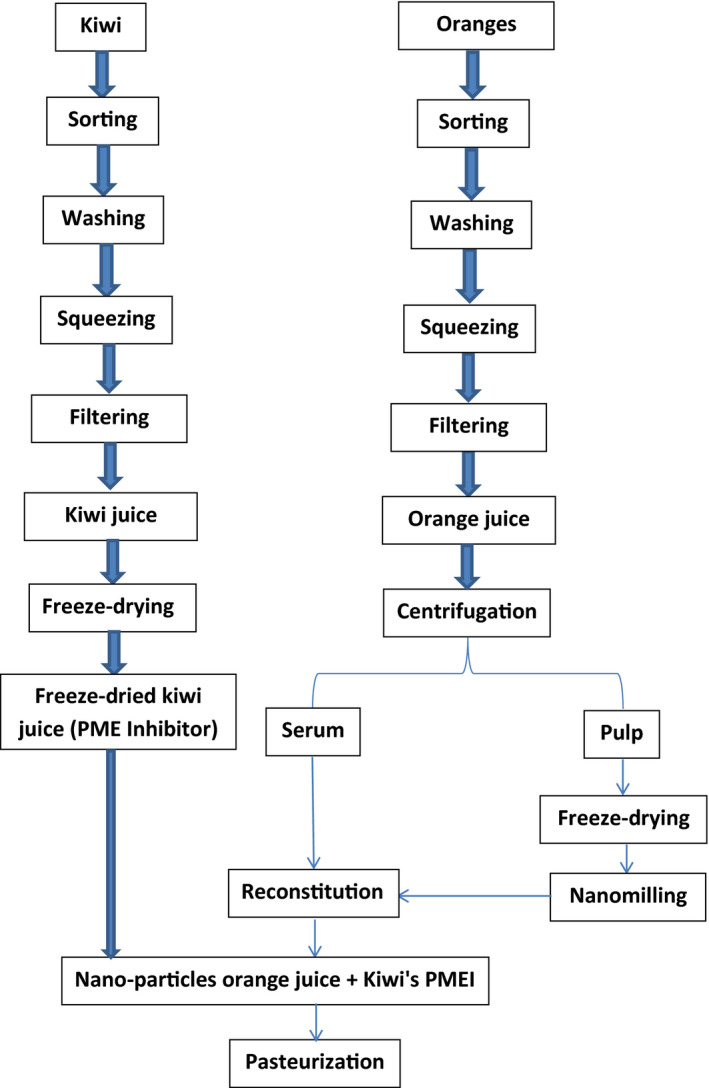
Flow chart of Nano‐particles orange juice processing

The described process (production of NPOJ with added PMEI from kiwi) is novel, simple and resulted in high‐quality OJ with the need to minimal heat treatments. It is believed that such process (Figure 6) is highly applicable and will be of value to small enterprises of food industry. However, a feasibility study is needed to evaluate the process.

## 4 CONCLUSIONS

PMEI from mature kiwi fruits proved to be a potent inhibitor for PME in OJ. Such inhibitor resulted in the inhibition of almost 90% of PME activity in OJ without affecting the microbial or the sensory quality of the juice. Nanomilling was successfully utilized for the first time in the preparation of NPOJ. This juice showed better cloud stability as well as microbial quality. However, a relatively higher degree of bitterness was obtained. The combined use of PMEI and nanomilling successfully allowed to use a lower pasteurization temperature (60°C/5 min) than those at 90’s°C needed to inactivate the highly thermostable PMEs. Such milder treatment resulted in a better‐quality juice with comparable characteristics as control and with accepted shelf life of 3 weeks.

## CONFLICT OF INTEREST

The authors declare no conflict of interests.

## ETHICAL STATEMENT

This study does not involve any human or animal testing.
